# Tumor Location Influences Oncologic Outcomes of Hepatocellular Carcinoma Patients Undergoing Radiofrequency Ablation

**DOI:** 10.3390/cancers10100378

**Published:** 2018-10-10

**Authors:** Jinbin Chen, Kangqiang Peng, Dandan Hu, Jingxian Shen, Zhongguo Zhou, Li Xu, Jiancong Chen, Yangxun Pan, Juncheng Wang, Yaojun Zhang, Minshan Chen

**Affiliations:** 1 State Key Laboratory of Oncology in South China, Collaborative Innovation Center for Cancer Medicine, Sun Yat-sen University Cancer Center, Sun Yat-sen University, Guangzhou 510060, Guangdong, China; chenjb@sysucc.org.cn (Jin.C.); pengkq@sysucc.org.cn (K.P.); hudd@sysucc.org.cn (D.H.); shenjx@sysucc.org.cn (J.S.); zhouzhg@sysucc.org.cn (Z.Z.); xuli@sysucc.org.cn (L.X.); chenjc@sysucc.org.cn (Jian.C.); panyx@sysucc.org.cn (Y.P.); wangjch@sysucc.org.cn (J.W.); 2 Department of Hepatobiliary Oncology, Sun Yat-sen University Cancer Center, Sun Yat-sen University, Guangzhou 510060, Guangdong, China; 3 Department of Radiology, Sun Yat-sen University Cancer Center, Sun Yat-sen University, Guangzhou 510060, Guangdong, China

**Keywords:** hepatocellular carcinoma, radiofrequency ablation, recurrence, tumor location, prognosis

## Abstract

Radiofrequency ablation (RFA) is recommended as a first-line therapy for small hepatocellular carcinoma (HCC). Tumor location is a potential factor influencing the procedure of RFA. To compare oncologic outcomes of RFA for different tumor locations, this retrospective study enrolled 194 patients with small HCC who had undertaken RFA. The HCC nodules were classified as peri-hepatic-vein (pHV) or non-pHV, peri-portal-vein (pPV) or non-pPV, and subcapsular or non-subcapsular HCC. The regional recurrence-free survival (rRFS), overall survival (OS), recurrence-free survival (recurrence in any location, RFS) and distant recurrence-free survival (dRFS) were compared. Operation failures were recorded in five pPV HCC patients, which was more frequent than in non-pPV HCC patients (*p* = 0.041). The 1-, 3-, and 5-year rRFS was 68.7%, 53.7%, and 53.7% for pHV patients and 85.1%, 76.1%, and 71.9% for non-pHV patients, respectively (*p* = 0.012). After propensity score matching, the 1-, 3-, and 5-year rRFS was still worse than that of non-pHV patients (*p* = 0.013). The OS, RFS, and dRFS were not significantly different between groups. Conclusions: A pHV location was a risk factor for the regional recurrence after RFA in small HCC patients. The tumor location may not influence OS, RFS, and dRFS. Additionally, a pPV location was a potential high-risk factor for incomplete ablation.

## 1. Introduction

Liver cancer is one of the most common malignancies worldwide, with an estimated 782,500 new liver cancer cases and 745,500 deaths having occurred worldwide in 2012 [1]. Hepatocellular carcinoma (HCC) is the most frequent primary liver malignancy. Radiofrequency ablation (RFA) has been recognized as a minimally invasive procedure that is as comparably effective as liver resection for small HCCs, and it was also recommended as a first-line therapy for small HCCs [2,3,4]. 

Tumor location is a potential factor influencing the procedural success of RFA treatment [5,6]. Being close to a large vessel or the liver capsule is potentially a high-risk location for RFA. A large vessel may drag thermal energy away during RFA targeting a perivascular tumor. This effect is called heat-sink effect and is regarded as a high-risk factor for incomplete ablation. A subcapsular location increases the difficulty of placing an electrode. Furthermore, to avoid bleeding and injuring the adjacent organs, the ablation area along the liver capsule is limited. Whether a high-risk location will affect the recurrence and overall survival is still controversial [5,6,7,8,9]. 

Furthermore, there are two vein systems of the liver, namely, the hepatic vein system and the portal vein system. It remains unclear if the relationship between the tumor and either one of the vein systems leads to different outcomes of the RFA procedure. Kang et al. compared the therapeutic outcomes of RFA for small perivascular HCC and non-perivascular HCC and drew the conclusion that the outcomes for small perivascular HCC were similar to those for non-perivascular HCC [7]. However, no further comparison was made to evaluate the difference stratified by portal vein and hepatic vein systems. 

Thus, the aim of our study is to compare the tumor control and the survival outcomes of RFA for different tumor locations relative to vessels and liver capsular for a single small HCC. 

## 2. Results

### 2.1. Baseline Characteristics

**Figure 1:**
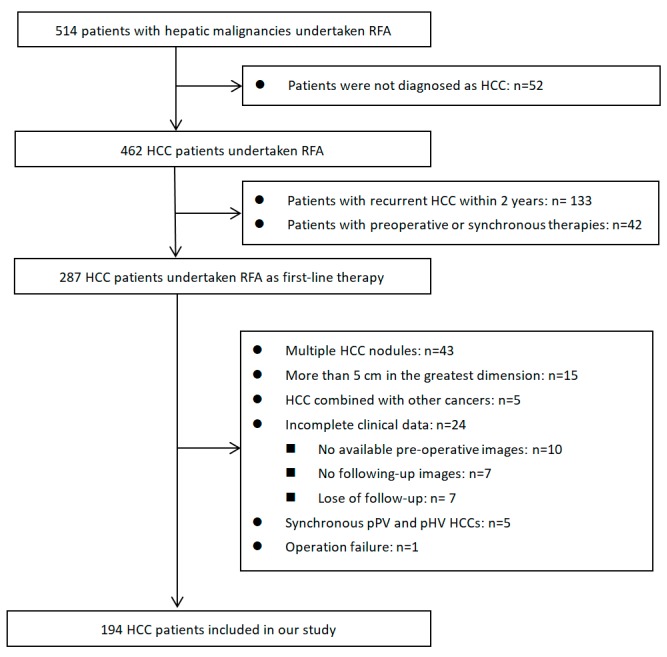
Flow diagram of patients identified, included, and excluded. RAF: Radiofrequency ablation; HCC: Hepatocellular carcinoma.

A total of 514 patients with hepatic malignancies undertook RFA in our center between January 2010 and December 2014. Among them, 194 cases were eligible and ultimately included in our study (inclusion and exclusion criteria are shown in Figure 1). The baseline characteristics of the whole cohort are shown in Table 1. The mean age of the patients was 55.60 years old. Most of them were male (162/194, 83.5%) and had hepatitis B virus (HBV) infection (163/194, 84.0%). Almost all patients had preserved liver function (Child–Pugh Class A, 189/194, 97.4%). There were 154 patients initially diagnosed as HCC, and the remaining 40 patients were diagnosed with recurrent HCC 2 years or longer after initial radical treatments. The HCC nodules were smaller than 3 cm in 156 patients (156/194, 80.4%) and between 3 and 5 cm in 38 patients (38/194, 19.6%). Liver cirrhosis was diagnosed in 117 patients. According to the tumor locations, there were 38 peri-portal-vein (pPV) HCCs (19.6%), 45 peri-hepatic-vein (pHV) HCCs (23.2%), and 65 subcapsular HCCs (33.5%). 

After being assigned to different groups according to tumor location, the baseline characteristics were analyzed between groups. All baseline characteristics were comparable between the pPV HCC group and the non-pHV HCC group (Table 1). The same results were obtained with subcapsular HCC patients and non-subcapsular HCC patients. However, a higher proportion of female was recorded in the pPV group than in non-pPV group (11/38, 28.9% in pPV group vs. 21/156, 13.5% in non-pPV group, *p* = 0.021). Moreover, 26.3% of pPV patients had a serum alpha-fetoprotein (AFP) level ≥ 400 ng/mL, which is higher than that of non-pPV patients (20/156, 12.8%, *p* = 0.047). More RFA cycles were recorded in pPV patients than in non-pPV ones (1.65 vs. 1.41 cycles, *p* = 0.025).

### 2.2. Operation Success and Complications

There were 11 failed cases (5.67%, 11/194) of the RFA operation recorded according to the radiological result 1 month after the first procedure (Table 1). Operation failures were recorded in five pPV HCC patients, which was more frequent than in non-pPV HCC patients (5/33, 15.2% in pPV group vs. 6/151, 4.0% in non-pPV group, *p* = 0.041, Table 1). Ten of these patients received another session of RFA, and the HCC nodules were finally ablated completely. One patient received liver resection to remove the incompletely ablated tumor. Only one case of major complication was recorded. The patient with severe cirrhosis suffered impaired liver and renal function after the RFA operation, and their postoperative hospital stay was prolonged to 9 days.

### 2.3. Recurrence and Survival Outcomes

The median follow-up period was 52.3 months, ranging from 6.1 to 107.3 months. To the date of the last follow-up, 49 cases of death, 118 cases of recurrence, 98 cases of distant recurrence, and 59 cases of regional recurrence were recorded. For the whole cohort, the 1-, 3-, and 5-year regional recurrence-free survival (rRFS) was 81.2%, 70.8%, and 67.6%. The 1-, 3-, and 5-year overall survival (OS) was 98.4%, 86.6%, and 74.8%, respectively. The corresponding recurrence-free survival (recurrence in any location, RFS) and distant recurrence-free survival (dRFS) were 69.7%, 44.9%, and 31.9% and 78.1%, 56.5%, and 40.8%, respectively.

**Figure 2:**
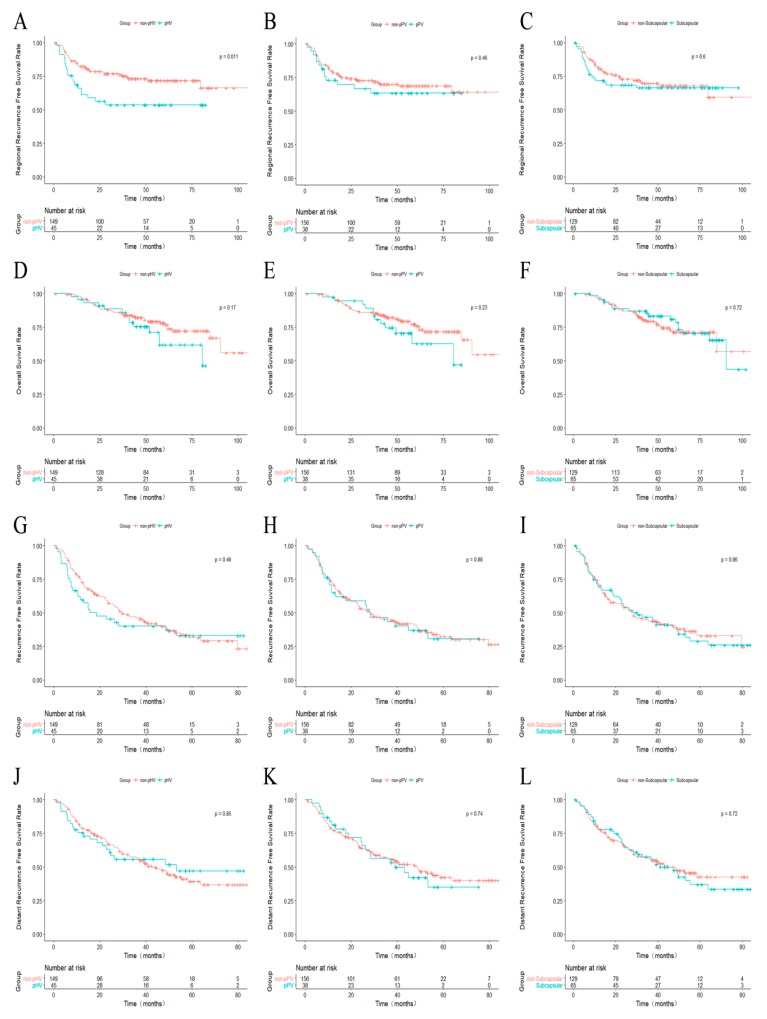
Kaplan–Meier curves comparing recurrence and overall survival from different groups. (**A**–**C**) Comparing rRFS between non-pHV and pHV (**A**), non-pPV and pPV (**B**), subcapsular and non-subcapsular (**C**) HCCs; (**D**–**F**) comparing OS between non-pHV and pHV (**D**), non-pPV and pPV (**E**), subcapsular and non-subcapsular (**F**) HCCs; (**G**–**I**) comparing RFS between non-pHV and pHV (**G**), non-pPV and pPV (**H**), subcapsular and non-subcapsular (**I**) HCCs; (**J**–**L**) comparing dRFS between non-pHV and pHV (**J**), non-pPV and pPV (**K**), subcapsular and non-subcapsular (**L**) HCCs. pHV: peri-hepatic vein; pPV: peri-portal-vein; HCC, hepatocellular carcinoma; rRFS: regional recurrence-free survival; OS: overall survival; RFS: recurrence-free survival; dRFS, distant recurrence-free survival.

Regional recurrence was recorded in 20 pHV HCC patients (20/45, 44.4%) and 39 non-pHV patients (39/149, 26.2%) (*p* = 0.020). The 1-, 3-, and 5-year rRFS was 85.1%, 76.1%, and 71.9% for non-pHV patients and 68.7%, 53.7%, and 53.7% for pHV patients, respectively (*p* = 0.012, Figure 2A). The 1-, 3-, and 5-year rRFS was 83.2%, 72.7%, and 68.8% for non-pPV patients and 73.1%, 63.4%, and 63.4% for pPV patients, respectively (*p* = 0.463, Figure 2B). For the non-subcapsular patients, the 1-, 3-, and 5-year rRFS was not significantly different from subcapsular patients (85.2%, 72.0%, and 68.0% vs. 73.4%, 68.4%, and 66.4%, *p* = 0.598, Figure 2C).

The survival outcomes were compared between pPV HCC patients and non-pPV HCC patients (Figure 2D–F). The 1-, 3-, and 5-year OS was 98.7%, 86.0%, and 77.2% for non-pPV HCC patients and 97.40%, 89.10%, and 62.80% for pPV HCC patients (*p* = 0.231). No significant difference was identified. Similarly, the difference between non-pHV HCC patients and pHV HCC patients was not significant (98.6%, 86.0%, and 78.2% vs. 97.8%, 88.5%, and 61.9%, *p* = 0.171). For the non-subcapsular HCC patients, the 1-, 3-, and 5-year OS was 98.4%, 86.4%, and 71.3%, respectively. For subcapsular HCC patients, these values were 98.4%, 87.0%, and 81.0%. There was no significant difference between these two groups (*p* = 0.720).

When comparing the RFS and dRFS between groups (pPV HCC vs. non-pHV HCC; pHV HCC vs. non-pHV HCC; subcapsular vs. non-subcapsular HCC), no significant difference was identified (Figure 2G–L).

### 2.4. Univariate and Multivariate Analysis

As shown in Table 2, for rRFS, univariate analysis results showed that only the pHV HCC was significant (HR: 1.98, 95% CI: 1.15–3.40, *p* = 0.013). For RFS, the only factor identified was AFP levels (HR: 1.54, 95%CI: 1.03–2.30, *p* = 0.037). For dRFS, the only factor identified was a higher alanine aminotransferase (ALT) level (HR: 1.67, 95% CI: 1.12–2.49, *p* = 0.012). Multivariate analysis was not performed since only one factor was identified in the univariate analysis.

The univariate analysis performed to determine the baseline prognostic factors for OS showed that an age older than 55 years, liver cirrhosis, splenomegaly, and higher Child–Pugh scores were statistically significant (*p* < 0.05, Table 2). A multivariate analysis with the use of a Cox proportional hazards model, which included all the significant factors from the univariate analysis, identified two baseline characteristics that were prognostic indicators of OS: older than 55 years (HR: 2.00, 95% CI: 1.08–3.69, *p* = 0.028) and higher Child–Pugh scores (HR: 2.67, 95% CI: 1.70–4.19, *p* < 0.001, Table 3).

### 2.5. Further Analysis for pHV and Non-pHV HCCs

**Figure 3:**
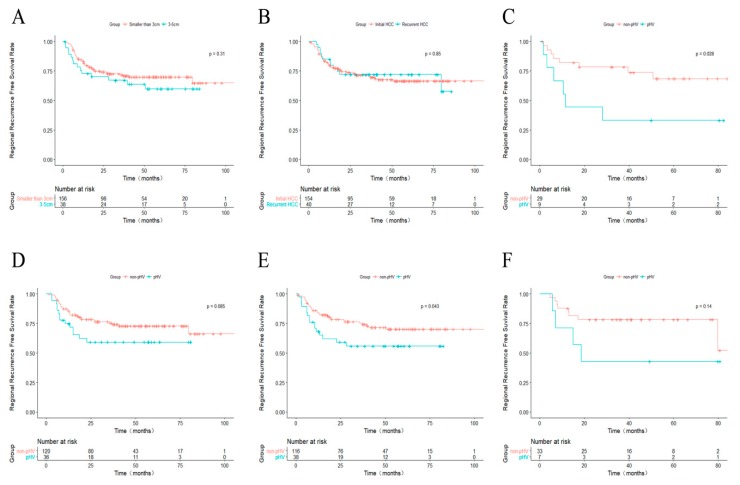
Kaplan–Meier curves comparing rRFS. (**A**) between HCCs smaller than 3 cm and 3–5 cm; (**B**) between initially diagnosed HCCs and recurrent HCCs; (**C**–**F**) between non-pHV and pHV HCCs in stratified subgroups; (**C**) HCCs between 3 and 5 cm; (**D**) HCCs smaller than 3 cm; (**E**) initially diagnosed HCCs; (**F**) recurrent HCCs. rRFS: regional recurrence-free survival; HCC: hepatocellular carcinoma; pHV: peri-hepatic vein.

The differences in rRFS for different tumor sizes and tumor types (initially diagnosed HCC or recurrent HCC) were evaluated. The 1-, 3-, and 5-year rRFS for patients with HCC ≤3.0 cm was 83.2%, 71.8%, and 69.9%, respectively (Figure 3A), and the corresponding rRFS was 73.0%, 67.2%, and 59.9% for those with HCC 3.1–5.0 cm. Even though the *p* value was larger than 0.05, an obvious curve separation could be observed. The difference in rRFS between the initial HCC and the recurrent HCC was not significant (Figure 3B). We also performed stratified subgroup survival analyses for rRFS according to the tumor size and whether the lesion was a recurrent HCC. In the subgroup with HCC 3.1–5.0 cm, the rRFS of pHV HCC patients was significantly lower than the rRFS of non-pHV ones (*p* = 0.028, Figure 3C). Likewise, for patients with HCC ≤3.0 cm, the rRFS was shorter than in non-pHV ones (Figure 3D). However, the *p* value was 0.085, though an obvious separation of the Kaplan–Meier curves was observed. Similarly, in the subgroup with initial HCCs, the rRFS of pHV HCC patients was significant shorter than the rRFS of non-pHV ones (*p* = 0.043, Figure 3E). However, the difference was not significant in the subgroup with recurrent HCCs (*p* = 0.141, Figure 3F). 

**Figure 4:**
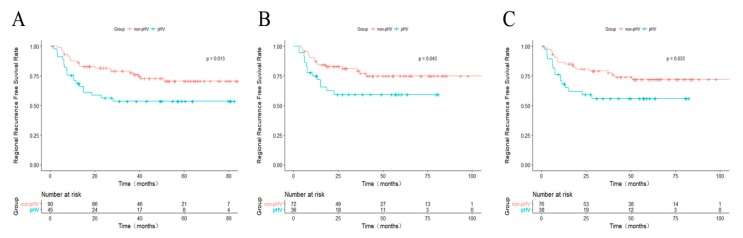
Kaplan–Meier curves comparing rRFS between non-pHV and pHV HCC patients after propensity score-match. (**A**) For all cases; (**B**) For HCCs smaller than 3 cm; (**C**) For initially diagnosed HCCs. rRFS: regional recurrence-free survival; HCC: hepatocellular carcinoma; pHV: peri-hepatic vein.

A 1:2 propensity score-matched analysis was performed to confirm the results above. A total of 45 pHV and 90 non-pHV HCC patients were matched. The 1-, 3-, and 5-year rRFS were 84.3%, 77.7%, 70.8% for non-pHV patients and 68.7%, 53.7%, 53.7% for pHV patients (*p* = 0.013, Figure 4A). For the subgroup of patients with HCC ≤3.0 cm and patients with initially diagnosed HCC, the 1:2 propensity score-matched analyses were also performed. And the results showed that the rRFS was better for the non-pHV HCC patients in both groups (Figure 4B,C).

## 3. Discussion

In the present retrospective study, we evaluated the roles of tumor location relative to liver capsule and large vessels in the oncologic outcomes after RFA treatment. More operation failures were recorded in pPV HCC patients after the first procedure. Additionally, the rRFS was worse for the pHV HCC patients than non-pHV HCC patients. However, the OS, RFS, and dRFS were not significantly different between groups. We may conclude that the tumor location close to a large portal vein was a potential high-risk factor for incomplete ablation, while being close to a hepatic vein was a risk factor for regional recurrence, but neither of these locations contributes to a significant decrease of OS.

RFA was recommended as one of the first-line therapies for small HCC [10]. The long-term overall survival of small HCC patients who received RFA was confirmed equivalent to those who received liver resection [2,11,12]. However, a worse tumor control rate after RFA treatment when compared with liver resection was reported, especially for tumors located at specific sites of the liver [11,13,14]. Potential explanations are that the thermal ablation region is relatively limited and micrometastases are more likely to escape the thermal effect. Furthermore, incomplete ablation may promote rapid progression of the residual tumor [15].

Thermal energy is the basis of RFA treatment [16]. The factors influencing thermal energy production and dissipation decide the treatment outcomes indirectly, and the ablation systems may also influence the tumor control [17]. In previous studies, subcapsular and perivascular nodules were regarded as predictors for worse tumor control. For the subcapsular HCCs, adjacent organs should be well protected and excessive thermal energy must be avoided, making complete ablation more difficult to achieve. Komorizono et al. drew the conclusion that subcapsular location was independently associated with local recurrence with a risk ratio of 5.5 [6]. However, a recent study reported that the survival and tumor control outcome were similar between subcapsular and non-subcapsular HCC patients who received RFA as a first-line therapy [8]. The results of our study were similar to the latter. Furthermore, recurrent HCCs were also included in our study, and the results were consistent with those of newly diagnosed HCCs. 

For the perivascular HCCs, the heat-sink effect would make complete ablation more difficult. However, whether the vessels contiguous to the HCC nodules reduce the complete ablation ratio is still controversial. Lu et al. compared the short-term outcome between 74 non-perivascular tumors and 31 perivascular tumors and concluded that the presence of vessels at least 3 mm in size contiguous to hepatic tumors is a strong independent predictor of incomplete tumor destruction by RFA [5]. In another study enrolling 241 small HCC patients conducted by Kang et al., the long-term therapeutic outcomes of RFA, including local tumor progression rate, RFS, and OS, were similar to those for non-perivascular HCC [7]. However, these studies did not classify the types of vessels and the recurrence. In our study, the long-term outcomes were also analyzed. Furthermore, as we classified the large vessels into portal vein and hepatic vein, the perivascular HCCs were categorized into two groups: pPV HCCs and pHV HCCs. Meanwhile, intrahepatic recurrence was categorized into regional recurrence around the treatment area and intrahepatic distant recurrence based on the distinct mechanism of pathogenesis. Through more detailed comparison, we made an effort to evaluate the role that peri-tumor vessels played in the RFA procedure and outcomes. As the results showed, the rRFS in pHV HCC patients was worse than that in non-pHV patients, while the OS in pPV HCC and pHV HCC patients was not significantly different with non-pPV and non-pHV patients. 

In our study, a pPV location was a potential high risk for incomplete ablation, and the rRFS was worse in the pHV patients. In the univariate analysis, the only risk factor for rRFS identified was pHV HCC (HR: 1.98, 95% CI: 1.15–3.40, *p* = 0.013). To some extent, regional recurrence may result from the incomplete ablation because of the heat-sink effect. Sheiman et al. reported that the hepatic vein volume in contact with the tumor was correlated with heat capacity during RFA and further resulted in local recurrence [18]. 

A higher operation failure rate was recorded in pPV patients. The potential reasons are as follows. Firstly, the heat-sink effect made complete ablation more difficult. Secondly, the portal vein in the liver was accompanied by the bile duct in Glisson’s capsule. RFA-induced Glisson’s capsule-associated complications may have higher risks of poor prognosis [19]. In these cases, to avoid severe complications, such as bile duct injury, the ablation power is mild, and the operation time is not too long. Therefore, thermal ablation is limited, to some extent, and an incompletely ablated area is left. When operation failures are recorded, an additional session of RFA is performed. The second session of RFA could accomplish complete ablation and reduce the local recurrence rate during a longer follow-up period. That may be the reason the rRFS for pPV HCC patients did not differ from that of non-pPV HCC patients in our study.

Furthermore, residual tumor nodules after ablation may be detected by axial computed tomography (CT) or magnetic resonance imaging (MRI) scan 1 month after the procedure. Even though the regional recurrence was more frequent for pHV patients, no significant difference in OS was indicated when compared with non-pHV patients. Under regular follow-up, the regional recurrent tumor could be detected at an early stage, and a radical operation could be performed. The second-line radical treatments, such as RFA and liver resection, could also contribute to local tumor control. Tumor size is an important prognostic factor for postoperative recurrence and OS [20,21]. Tumors ≤3 cm have been recommended as being suitable for RFA [22,23]. As the result of subgroup analysis showed, pHV location was a risk factor for regional recurrence for patients with tumors 3.1–5.0 cm. The difference was not significant in the subgroup with nodules ≤3 cm. 

There were several limitations to our study. First, as a retrospective study, the choice of therapy was decided by the individual clinician’s experience, causing potential bias that could hardly be assessed. Second, this is a single-center study, and the number of pHV and pPV HCC patients was relatively small. To generalize this conclusion, a large cohort multicenter clinical trial is necessary. Third, both CT and MRI were used to classify the tumor location relative to the vessels and liver capsule. The difference between CT and MRI images may slightly influence the image outcomes.

## 4. Materials and Methods

### 4.1. Study Population and Design

This retrospective study enrolled 194 consecutive patients with small HCC who had undertaken RFA in the Department of Hepatobiliary Oncology of Sun Yat-sen University Cancer Center between January 2010 and December 2014. The study was approved by the institutional review board (IRB) of Sun Yat-sen University Cancer Center (approval number: B2018-032-01, 23 May 2018). At the time of submission, written and informed consent was obtained from the participants or their parent or legal guardian. All treatments were performed in accordance with relevant guidelines and regulations.

Inclusion criteria: (1) Diagnosed with HCC based on the criteria of the European Association for the Study of the Liver [24]. Pathological diagnosis was only required if the clinical diagnosis was not clear; (2) single nodular HCC that was 5 cm or smaller in the greatest dimension on CT or MRI scan; (3) initially identified or recurrent HCC detected 2 years or longer from the initial curative therapy; (4) RFA was selected as the first-line therapy; (5) Eastern Cooperative Oncology Group (ECOG) performance status of 0–1 and Child–Pugh Class A or B.

Exclusion criteria: (1) Unclear diagnosis or any evidence for tumor lesions other than the targeted HCC nodule; (2) Any preoperative or synchronous treatment, such as preoperative transcatheter arterial chemoembolization (TACE), ethanol injection, or systemic therapy; (3) HCC combined with other cancers; (4) Tumor in contact with both portal vein and hepatic vein; (5) RFA operation failure; (6) Incomplete clinical data.

### 4.2. Definition of Perivascular HCC and Subcapsular HCC

Based on their relation to large vessels and liver capsule, the HCC nodules were classified according to different criteria on the basis of CT or MRI findings [8,25]. HCCs were classified by two experienced radiologists (KQ Peng and JX Shen) independently who both had more than 10 years of experience in radiological diagnosis and were blind to clinical outcomes.

According to their relation to portal veins, the HCCs were classified as peri-portal-vein HCCs (pPV HCC) and non-peri-portal-vein HCCs (non-pPV HCC). The pPV HCC was defined as an index tumor with any contact with first- or second-degree branches of a portal vein that are 3 mm or greater in axial diameter. Otherwise, it was a non-pPV HCC. Similarly, peri-hepatic-vein (pHV) HCC and non-pHV HCC were classified according to their relation to hepatic veins. Subcapsular HCC was defined as an index tumor that abutted the liver capsule on axial or coronal CT and/or MRI (distance from the hepatic capsule to the tumor margin ≤1 mm). Otherwise, it was a non-subcapsular HCC.

### 4.3. RFA Procedure

All RFA operations were performed by MS Chen, an experienced oncologist who had more than 20 years of experience in RFA operations. The RFA procedure was described in our previous research [2,26]. Summarily, RFA was performed by using a commercially available system (RF 2000; Radio-Therapeutics, Mountain View, CA, USA) under real-time ultrasound guidance (EUB-2000; Hitachi Medical Systems, Tokyo, Japan) and a needle electrode with a 15-Ga insulated cannula with 10 hook-shaped expandable electrode tines with a diameter of 3.5 cm at expansion (LeVeen; Radio Therapeutics). A 15-G RFA needle was first inserted into the tumor. After the 10 tines of the needle were deployed, the RF generator was activated and initiated with 10 W of power. The power was increased 10 W per minute to 90 W. RFA was applied until either there was a marked increase in impedance or 15 min had elapsed. If a marked increase in impedance was not achieved, a second application of RFA was given. No more than three applications of RFA were given in a treatment session. For tumors ≤3.0 cm at their greatest dimension, a single ablation was performed. For tumors more than 3.0 cm at their greatest dimension, multiple overlapping ablations were performed.

### 4.4. Following Up

All patients underwent CT or MRI examination 1 month after the RFA operation to assess the technical success of the procedure. Successful RFA operation was defined as complete ablation and no enhanced tumor, as confirmed by CT or MRI scan. If any residual tumor was confirmed, an operation failure was recorded. A second session of RFA aiming at complete ablation was allowed. The date of the second session was recorded as the successful operation date. If resection or other therapies were undertaken after the first RFA operation failure, the cases were excluded from further analysis.

The patients were followed up 1 month after the initial treatment and every 3 months thereafter. Surveillance included routine blood test, liver function profile, AFP level analysis, and dynamic CT or MRI. CT of the chest, bone scintigraphy, or positron emission tomography CT (PET-CT) was performed when extra-hepatic metastases were suspected.

### 4.5. Outcomes

The primary outcome of this study was the rRFS, which was defined by the interval from the date of the successful RFA operation to the date of recurrence at the margin of the ablation zone or the date of the last follow-up. The secondary outcomes were OS, RFS, and dRFS. The definition of OS was the interval from the successful operation date to death by any cause or to the last follow-up date. RFS was defined by the interval from the date of the successful RFA operation to the date of recurrence in any location or the date of the last follow-up. Finally, dRFS was defined by the interval from the date of the successful RFA operation to the date of recurrence outside of the ablation area or the date of the last follow-up.

Complications were assessed according to the guidelines of the Society of Interventional Radiology [27]. The definition of major complication is an event that leads to substantial morbidity and disability resulting in hospital admission, or substantially lengthens the hospital stay. All other complications are considered minor. The duration of the postoperative hospital stay was also recorded.

### 4.6. Statistical Analysis

Statistical analysis was performed with SPSS 20.0 software (SPSS, Chicago, IL, USA) and R 3.4.3 (http://www.r-project.org/). Student’s *t*-test was used to compare continuous variables when the data were distributed normally. The Mann–Whitney U test was used to compare skewed data, and the chi-square or Fisher’s exact test was used for categorical variables. OS, RFS, rRFS, and dRFS were estimated by Kaplan–Meier analysis, and the curves were compared by the log-rank test. The Cox proportional hazards model was used for multivariate analysis of survival. All variables found significant in the univariate analysis were included in the multivariate model. To confirm the result from the whole cohort, a propensity score-matched analysis was performed for 1:2 match through the nearest available matching using the “MatchIt” package of R. The following variables were matched for Child–Pugh scores: tumor size level, recurrent HCC or not, and liver cirrhosis. A two-sided *p* value < 0.05 was considered to indicate statistical significance. 

## 5. Conclusions

In this retrospective study investigating the influence of tumor location on RFA treatment outcomes, the results indicate that a pHV location was a risk factor for regional recurrence in small HCC patients. The tumor location may not influence OS, RFS, and dRFS for small HCC patients. We also identified a pPV location as a potential high-risk factor for incomplete ablation. We are expecting further prospective randomized studies to confirm our conclusions.

## Figures and Tables

**Table 1:** Baseline characteristics.

Variables	Non-pHV	pHV	*p*	Non-pPV	pPV	*p*	Non-Subcapsular	Subcapsular	*p*
Case Number	149	45		156	38		129	65	
Age (years)	55.05 ± 12.54	57.42 ± 12.61	0.269	56.04 ± 12.44	53.82 ± 13.06	0.329	54.40 ± 12.33	57.98 ± 1.47	0.061
Gender (Male/female)	123/26	39/6	0.514	135/21	27/11	0.021 *	108/21	54/11	0.909
HBV Infection (Y/N)	123/26	40/5	0.309	130/26	33/5	0.597	109/20	54/11	0.799
HCV Infection (Y/N)	8/141	1/44	0.688	8/148	1/37	1.000	6/123	3/62	1.000
Child-Pugh (A/B)	147/2	42/3	0.083	152/4	37/1	1.000	124/5	65/0	0.171
AFP (<400/≥400, ng/mL)	125/24	39/6	0.652	136/20	28/10	0.047 *	111/18	53/12	0.412
Recurrent HCC (Y/N)	33/116	7/38	0.338	36/120	4/34	0.086	22/107	18/47	0.084
Tumor Size (<3/3–5 cm)	120/29	36/9	0.937	127/29	29/9	0.478	108/21	48/17	0.102
Cirrhosis (Y/N)	93/56	24/21	0.275	93/63	24/14	0.689	77/52	40/25	0.804
Splenomegaly (Y/N)	54/95	18/27	0.647	58/98	14/24	0.969	48/81	24/41	0.969
Postoperative Hospital Day	3.42 ± 2.71	3.11 ± 1.32	0.468	3.40 ± 2.68	3.13 ± 1.234	0.552	3.37 ± 2.84	3.29 ± 1.47	0.832
RFA Cycle ^†^	1.43 ± 0.60	1.53 ± 0.66	0.352	1.41 ± 0.58	1.65 ± 0.71	0.025 *	1.43 ± 0.60	1.52 ± 0.64	0.300
Operation Failure ^††^	8/142	3/42	0.718	6/151	5/33	0.041 *	8/122	3/62	0.755

* *p* < 0.05; ^†^ The number of applications of radiofrequency ablation (RFA) given in a treatment session; ^††^ Including the case received liver resection as rescued treatment which was excluded for further analysis. pHV: peri-hepatic vein; pPV: peri-portal-vein; HCC: hepatocellular carcinoma; HBV: hepatitis B virus; HCV: hepatitis C virus; RFA: radiofrequency ablation.

**Table 2:** Univariate analysis for prognostic factors.

Factor	OS		RFS		rRFS		dRFS	
	HR (95% CI)	*p*	HR (95% CI)	*p*	HR (95% CI)	*p*	HR (95% CI)	*p*
Older age (≥55 vs. <55, years)	1.94 (1.07–3.52)	0.030 *	0.87 (0.61–1.26)	0.468	0.79 (0.48–1.32)	0.372	1.02 (0.68–1.51)	0.938
Cirrhosis	2.26 (1.18–4.34)	0.014 *	1.14 (0.79–1.65)	0.488	1.13 (0.67–1.90)	0.654	1.13 (0.75–1.69)	0.567
Splenomegaly	2.15 (1.23–3.78)	0.008 *	0.97 (0.66–1.42)	0.886	0.93 (0.54–1.59)	0.780	1.06 (0.70–1.60)	0.798
Peri-hepatic-vein location	1.55 (0.83–2.89)	0.174	1.18 (0.77–1.80)	0.455	1.98 (1.15–3.40)	0.013 *	0.95 (0.59–1.55)	0.849
ALT level (≥35 vs. <35, U/L)	1.08 (0.60–1.93)	0.795	1.38 (0.96–2.00)	0.083	0.96 (0.56–1.64)	0.868	1.67 (1.12–2.49)	0.012 *
AFP level (≥400 vs. <400, ng/mL)	1.13 (0.53–2.40)	0.761	1.58 (1.00–2.49)	0.049 *	1.00 (0.49–2.03)	0.999	1.53 (0.92–2.52)	0.098
Child–Pugh score	3.47 (2.27–5.31)	<0.001 *	1.04 (0.66–1.64)	0.881	1.39 (0.83–2.33)	0.212	1.15 (0.71–1.86)	0.569
Size level (3–5 vs. <3, cm)	1.38 (0.73–2.60)	0.32	1.00 (0.63–1.57)	0.992	1.36 (0.74–2.47)	0.32	0.89 (0.53–1.49)	0.662
Recurrent tumor	0.77 (0.36–1.65)	0.503	1.38 (0.91–2.10)	0.127	0.93 (0.50–1.76)	0.834	1.31 (0.83–2.06)	0.248

* *p* < 0.05, OS: overall survival; RFS: recurrence-free survival; rRFS: regional recurrence-free survival; dRFS: distant recurrence-free survival; ALT: alanine aminotransferase; AFP: alpha-fetoprotein; HR: hazard ratio.

**Table 3:** Multivariate analysis for prognostic factors of overall survival (OS).

Factor	HR (95% CI)	*p* Value
Older age (≥55 vs. <55, years)	2.00 (1.08–3.69)	0.028 *
Cirrhosis	1.82 (0.88–3.74)	0.105
Splenomegaly	1.49 (0.77–2.85)	0.233
Child–Pugh score	2.67 (1.70–4.19)	<0.001 *

* *p* < 0.05.
